# Improving the Quality of Measurements Made by Alphasense NO_2_ Non-Reference Sensors Using the Mathematical Methods

**DOI:** 10.3390/s22103619

**Published:** 2022-05-10

**Authors:** Mariusz Rogulski, Artur Badyda, Anna Gayer, Johnny Reis

**Affiliations:** 1 Faculty of Building Services, Hydro and Environmental Engineering, Warsaw University of Technology, Nowowiejska 20, 00-653 Warsaw, Poland; artur.badyda@pw.edu.pl (A.B.); anna.gayer@gmail.com (A.G.); 2 CESAM—Center for Environmental and Marine Studies & Department Environment and Planning, University of Aveiro, 3810-193 Aveiro, Portugal; johnnydreis@ua.pt

**Keywords:** air quality, Alphasense NO_2_ sensors, comparative measurements, correction function, Alphasense NO2-B43F, electrochemical sensors, in-field nitrogen dioxide monitoring

## Abstract

Conventional NO_2_ monitoring devices are relatively cumbersome, expensive, and have a relatively high-power consumption that limits their use to fixed sites. On the other hand, they offer high-quality measurements. In contrast, the low-cost NO_2_ sensors offer greater flexibility, are smaller, and allow greater coverage of the area with the measuring devices. However, their disadvantage is much lower accuracy. The main goal of this study was to investigate the measurement data quality of NO2-B43F Alphasense sensors. The measurement performance analysis of Alphasense NO2-B43F sensors was conducted in two research areas in Poland. Sensors were placed near fixed, professional air quality monitoring stations, carrying out measurements based on reference methods, in the following periods: July–November, and December–May. Results of the study show that without using sophisticated correction methods, the range of measured air pollution concentrations may be greater than their actual values in ambient air—measured in the field by fixed stations. In the case of summer months (with air temperature over 30 °C), the long-term mean absolute percentage error was over 150% and the sensors, using the methods recommended by the manufacturer, in the case of high temperatures could even show negative values. After applying the mathematical correction functions proposed in this article, it was possible to significantly reduce long-term errors (to 40–70% per month, regardless of the location of the measurements) and eliminate negative measurement values. The proposed method is based on the recalculation of the raw measurement, air temperature, and air RH and does not require the use of extensive analytical tools.

## 1. Introduction

Air pollution is a complex problem posing multiple challenges in terms of management and mitigation of harmful pollutants [1].

Life quality and human health are affected by air pollution, especially in urban areas, where most of the population lives [2,3]. Europe’s most problematic pollutants in terms of health are PM, NO_2_, and ground-level O_3_ [4]. Nitrogen dioxide (NO_2_) is one of the major pollutant gases, and its emanation is mainly caused by traffic [5].

Enhancing the spatial and temporal resolution of air pollution monitoring is nowadays one of the emerging challenges [6]. With the development of low-cost air quality sensors lot of research has been focused on achieving accurate, robust, and reliable air quality data [7,8]. The equipment was evaluated according to its performance in different environments, seasons, and meteorological conditions [9,10]. Critical issues faced by researchers are mostly correction methods [11,12] and the long-term stability of the sensors [13]. To characterize interferences and improve correction functions, both laboratory and ambient tests were conducted [14,15,16,17,18].

Bauerová et al. evaluated low-cost electrochemical sensors in field testing measurements [19]. The data from different sensors (for SO_2_, NO_2_, O_3_, and CO) were compared with co-located reference monitors used within the Czech National Air Quality Monitoring Network. The results showed that in addition to the given reduced measurement accuracy of the sensors, the data quality depends on the early detection of defective units and changes caused by the effect of meteorological conditions (effect of air temperature and humidity on gas sensors). This author concluded that comparative measurement is necessary before each sensor’s field application.

One of the biggest studies in comparative measurement focused on validating electrochemical (EC) sensor measurements of CO, NO, NO_2_, and O_3_ at an urban neighborhood site with pollutant concentration ranges: [NO_2_] = 11.7 ± 8.3 ppb and [O_3_] = 23.2 ± 12.5 ppb [20]. Through the use of high-dimensional model representation, the authors showed that interference effects derived from the variable ambient gas concentration mix and changing environmental conditions over three seasons can be effectively modeled for the Alphasense sensors. 

In other research, the authors calibrated samples of three NO2-B43F sensors and three OX-B431 sensors with NO_2_ and O_3_ exclusively and conducted mixture experiments over a range of 0–1.0 ppm NO_2_ and 0–125 ppb O_3_ to evaluate the ability of the paired sensors to quantify NO_2_ and O_3_ concentrations in the mixture [21]. Although the slopes of the response among samples of three sensors of each type varied by as much as 37%, the individual response of the NO2-B43F sensors to NO_2_ and OX-B431 sensors to NO_2_ and O_3_ were highly linear over the concentrations studied (R^2^ > 0.99). 

Many authors (e.g., mentioned below) suggest that to improve the quality of measurements, a mathematical correction is necessary; however, determining the correction functions remains relatively poorly presented [22]. Lin et al. examined O_3_, NO_2_ (Aeroqual), and particulate matter (RTI) sensors and determined simple, linear correction functions [23]. Additionally, the mutual influence of particular pollutants was analyzed. The study concluded that better results are obtained by the correction and the use of a data set from many sensors, instead of relying only on data from the sensor for which the correction function is determined and which were subjected to comparative measurements. In different research scenarios, after carrying out the measurements with the Alphasense NO_2_ electrochemical sensor and reference device and determining the correction function, the coefficients of determination for the tested set of sensors increased from the range of 0.3–0.7 to 0.6–0.9 [24]. The authors concluded that measurement campaigns using low-cost sensors based on modern generations of electrochemical NO_2_ sensors can provide useful complementary data on local air quality in an urban environment. In the study of Wei et al., electrochemical sensors (Alphasense B4 series) for carbon monoxide (CO), nitric oxide (NO), nitrogen dioxide (NO_2_), and oxidants (O_x_) were evaluated under controlled laboratory conditions to identify the influencing factors with sensor outputs [10]. Based on the laboratory tests, the authors developed different correction methods to compensate for the impact of ambient conditions.

**Figure 1:**
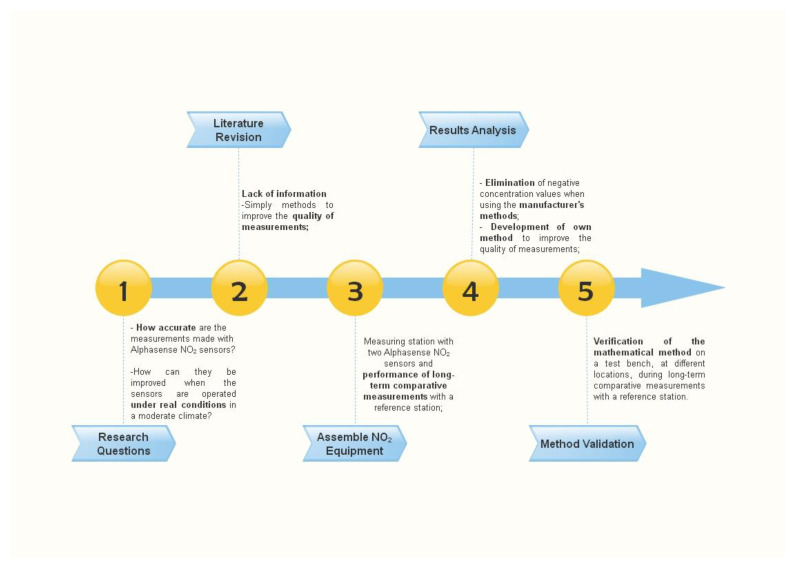
The research flowchart.

The main goal of this study was to investigate the measurement data quality of NO_2_ Alphasense (NO2-B43F) sensors. The research focused on interfering factors and lead to the calculation of expected errors in comparative measurements. Results showed that without improving correction methods, the range of measured air pollution concentrations may be greater than their actual values in ambient air. Therefore, based on the conducted comparative measurements with professional devices, the article proposes an innovative algorithm for converting raw measurements, taking into account air temperature and relative humidity. The proposed formula does not require complex analytical tools and a history of measurements, so it can be used in simple microcontrollers. Its effectiveness has been checked on measurements carried out in a location other than the original measurements. The research flowchart is presented in Figure 1.

## 2. Materials and Methods

Alphasense NO_2_ (NO2-B43F) sensors are popular, low-cost sensors for measuring the concentration of nitrogen dioxide in the ambient air using the electrochemical method (4-electrodes). The working principle of the NO_2_ electrochemical sensor is based on electrochemical reactions [5]. When the air passes through, it creates a reaction in the electrochemical cell. The surface of the working electrode is the site for the first half-reaction (oxidation), generating an electronic charge, balanced by the second half-reaction (reduction) that occurs at the counter electrode [25]. This type of sensor provides high selectivity, low limit of detection, low power consumption, and linear response to the target gas [26,27].

According to the manufacturer’s description, together with a dedicated electronic board, they enable the measurement of even small concentrations of nitrogen dioxide (ppb level). To test the sensors measuring devices were built. The main element was a small measuring chamber in which two Alphasense sensors were placed. To take the air directly from the surroundings, a small fan was installed at the inlet to the measuring chamber. The measuring chamber (with direct air intake from the environment) was placed in a larger housing, which contained electronic components necessary to power the sensors and microcontroller to acquire the results (voltages from electrodes acquired from the sensor’s transmitter board) and transfer them to the server. The inlet to the measuring chamber was directed downwards (analogous to the air outlet). As a result, the measuring chamber was not susceptible to wind force and rain/snow. 

**Figure 2:**
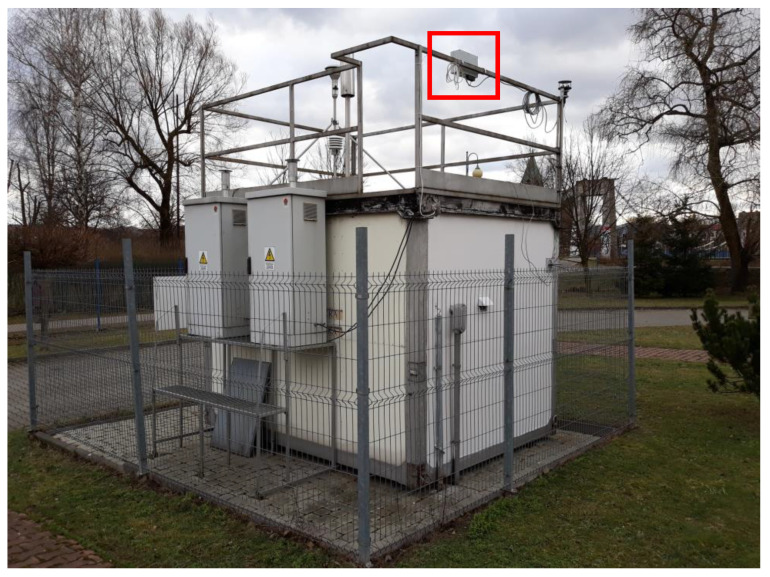
Measurement device with Alphasense sensors on the SEM station in Nowy Sącz.

**Figure 3:**
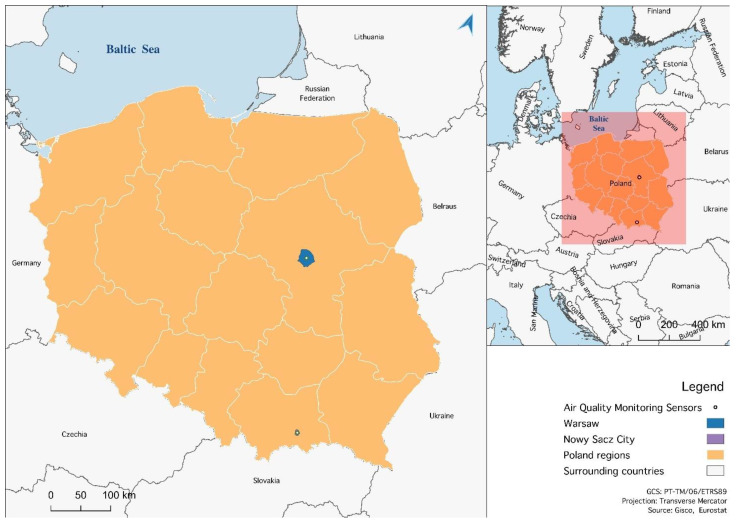
The locations of measurements.

The measurement performance analysis of Alphasense (NO2-B43F) sensors was conducted in two research fields. Sensors were placed at the air quality monitoring stations, carrying out measurements based on reference methods. The stations are part of the State Environmental Monitoring (SEM)—Figure 2. The locations and timing of comparative measurements were as follows (Figure 3):

- Nowy Sącz (a city in southern Poland) July–November 2019,
- Warszawa-Chrościckiego in Warsaw (central Poland) December 2019–May 2020.

From the research perspective, the city of Nowy Sącz is interesting due to its frequently occurring high air pollutant concentrations and varied weather conditions [28]. The town is situated in a diversified area—the lowest point of the city is located at an altitude of 272 m above sea level, and the highest point—475 m above sea level. There are typically urban areas with tenement houses, parks, and green areas as well as single-family houses. Some areas are supplied by the district heating network, while others, especially single-family houses, are equipped with individual boilers or fireplaces (mainly for solid fuels), being a key source of particulate matter emissions. On the other hand, Warsaw, which is the capital of Poland, has a slightly different climate but is also quite diverse in terms of the variability of pollutants and main sources of emission [29].

Alphasense sensors record the voltage related to the measured quantity at the two outputs of each sensor:

- WE_u_—voltage of the working electrode (mV);
- AE_u_—voltage of the auxiliary electrode (mV).

At the same time, the following values are provided individually for each sensor (as a result of calibration performed by the manufacturer):

- WE_e_—the value of the electronic offset for the used ISB (Alphasense Individual Sensor Board) for the working electrode (mV);
- AE_e_—the value of the electronic offset for the used ISB plate for the auxiliary electrode (mV);
- WE_0_—an indication of the working electrode in (mV) for air free of pollutants;
- AE_0_—an indication of the auxiliary electrode in (mV) in the case of unpolluted air;

and two parameters independent of a specific sensor:

- n_T_—factor provided by the manufacturer (correction depending on the temperature);
- k′_T_—factor provided by the manufacturer (correction depending on the temperature).

The values of the above-mentioned factors from the manufacturer’s documentation for the tested type of sensors are shown in Table 1. 

To convert the measured value to the pollutant concentration, depending on the sensor used, first, the corrected voltage value (WE_c_) is calculated and then the appropriate conversion factor (mV/ppb), also, given individually for each sensor, is applied. The last step is to convert the concentration to the appropriate unit (e.g., from (ppb) to (µg/m^3^)).

For the NO_2_-B43F sensors the manufacturer proposes the use of one of the following two equations to determine the corrected voltage (we will refer to them also as method (1) and method (2), respectively):

WE_C_ = (WE_U_ − WE_E_) − n_T_(AE_U_ − AE_E_), (1)

WE_C_ = (WE_U_ − WE_E_) − (WE_0_ − AE_0_) − k′_T_(AE_U_ − AE_E_), (2)

In the comparative measurements carried out at the monitoring station in Nowy Sącz, two NO_2_-B43F sensors were used. For each of these electrochemical sensors, called later in the text as NO2_1 and NO2_2, the following set of parameters was available: WE_E_, AE_E_, WE_0_, and AE_0_.

Measurement instruments incorporating these sensors recorded the values of the appropriate voltages every few seconds, and then, every minute relayed these data to the server. The data were aggregated, as a result of which, 1 h mean values were determined. The study includes the comparative analysis of the data obtained in this way and 1 h measurement data from SEM’s air quality monitoring station. The following statistical measures were used to compare the measurements from Alphasense sensors to measurements from the SEM station: Pearson’s correlation coefficient, mean error, mean percentage error, mean absolute error, mean absolute percentage error, and mean square error. All these statistical measures were determined for particular months of the measurement periods.

## 3. Results

In order to determine the quality of the obtained measurements, the measured voltage at both outputs of individual sensors was converted into concentrations with the use of both methods presented above. For the obtained results, the basic statistics for each month were calculated. The results are shown in Table 2. These statistical parameters were calculated by comparing the data from a given Alphasense sensor with the values obtained from the SEM measurement station.

Table 3 shows the average monthly temperature measured in the chamber of the device. It should be emphasized that the instrument was intentionally left unsheltered and therefore the maximum temperatures inside the device during the summer period often exceeded +40 °C.

The data presented in Table 2 show relatively different errors; in particular, months of the measurement period. For both tested sensors and both methods for converting voltages into concentrations suggested by the manufacturer, the lowest values of this factor were recorded in July. The best parameters were recorded in October. To some extent, this may have been related to the variability of actual concentrations. 

The highest absolute percentage error occurred for the measurements conducted in warm months. The decrease in the average temperature lowered the error. While in July, the absolute percentage error exceeded 130%; in November, it oscillated around 50%. 

A similar tendency is also visible in case of the absolute errors. The absolute errors decreased from 11–15 µg/m^3^ in July to 8–10 µg/m^3^ in November. It is also worth mentioning the high correlation of low-cost sensors’ indications—for the entire period, it was r = 0.952 after applying the method (1) and r = 0.949 after applying the method (2). This means that the readings of the sensors were repeatable and that they both reacted similarly to the parameters and changes in the atmospheric air, and the actual concentration values (and possibly other pollutants). This repeatability also implies the assumption that a single, universal method of mathematical correction of sensor indications can be created.

**Figure 4:**
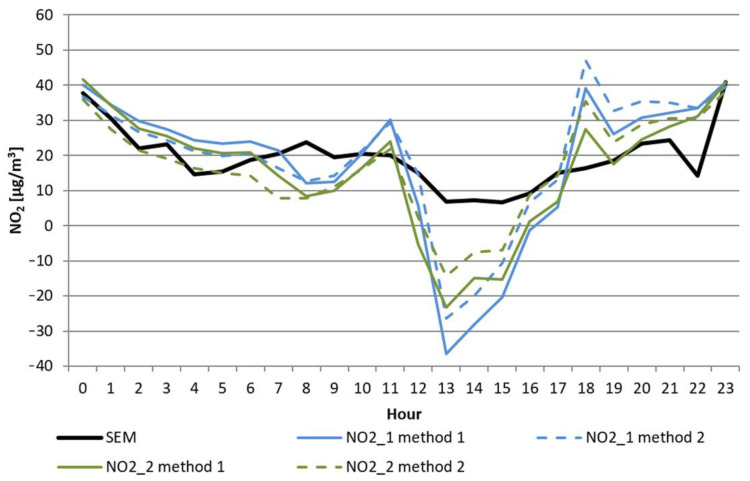
Daily variability of NO_2_ concentration in Nowy Sącz on 20 July 2019 was measured in the SEM station and calculated using two methods from the measurement results of two sensors (NO2_1 and NO2_2).

**Figure 5:**
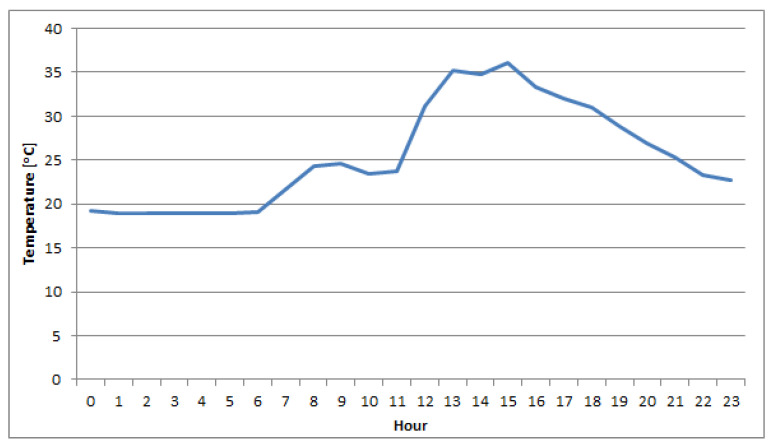
The daily temperature variability in Nowy Sącz on 20 July 2019 inside the measurement chamber.

During warm and hot days, when the temperature was approximately or exceeding 30 °C, both sensors regularly generated voltage that corresponded to negative concentrations of nitrogen dioxide after applying methods (1) or (2). The variability of 1 h mean concentrations of NO_2_ and the temperature during one of the study days are shown in Figure 4 and Figure 5.

Individually for both methods (1) and (2), deviations due to high temperature were corrected. After calculations, negative WE_C_ values and the relevant minimum T_WEC_ temperature were determined in this set. For method (1), it was T_WEC_ = 23.1 °C, while for method (2), T_WEC_ = 23.8 °C. Then, for all hours for which the average temperature was higher or equal to the determined minimum, the average temperature and the determined voltages WE_U_ and AE_U_ were collected. For each hour, the desired WE_C_° voltage was determined, which would correspond to the NO_2_ concentration measured in the SEM station. It means that the WE_C_° value denotes the result of the relationship (1) or (2) where the result from the Alphasense sensors would correspond exactly to the value determined by the SEM station. In the next step, the difference WE_C_′ = WE_C_° − WE_C_ (offset) was calculated, determined by how much the current indication from the low-cost sensor should be corrected to obtain the value corresponding to the measurement from the SEM.

Multiple regression was applied to the set of 1 h mean values (T, WE_U_, AE_U_, and WE_C_′), where the independent variables were: 1 h mean temperature (T), 1 h mean voltage WE_U_, and 1 h mean voltage AE_U_. The dependent variable was the WE_C_′ value.

As a result, for (1) and (2) the following relationships were obtained, respectively:

WE_C_′ = −3.489(WE_U_ − WE_E_) + 2.448(AE_U_ − AE_E_) + 0.103T + 3.39, (3)

WE_C_′ = −2.815(WE_U_ − WE_E_) + 2.118(AE_U_ − AE_E_) − 0.238T + 12.357, (4)

**Figure 6:**
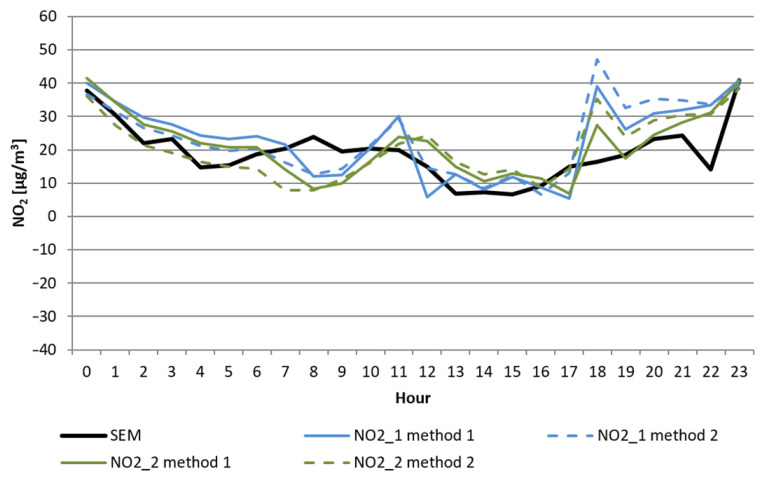
Daily variability of NO_2_ concentration in Nowy Sącz on 20 July 2019, after applying correction functions.

These additional WE_C_′ values were added to the WE_C_ for measurements where the T_WEC_ was higher than the predetermined cutoff for both methods. In the case of the previously presented data from 20 July 2019, the new volatility patterns are presented in Figure 6.

Equations (3) and (4) were added to (1) and (2), respectively, and again NO_2_ concentrations were recalculated for both Alphasense sensors. The results for July and August 2019 (being the months with the highest temperatures) are presented in Table 4.

The use of an offset (methods (3) or (4)) in most cases improved the quality of the results. First of all, the “negative” concentrations were removed, and the correlation with the measurements from the SEM stations was significantly improved. The highest improvement appeared in August, where correlation coefficients in relation to the SEM stations were r = 0.8. Correlation between NO2_1 and NO2_2 sensors in July were: r = 0.934 (for (3)) and r = 0.923 (for (4)), while in August: r = 0.959 (for (3)) and r = 0.946 (for (4)). The mean values of the absolute percentage errors and thus the absolute errors also improved significantly. In most cases, they were reduced by half. 

The next step was to analyze the set of measurement data and determine the new correction functions. First, the relationship between the Alphasense sensor indications and the measurements from the SEM stations was examined.

**Figure 7:**
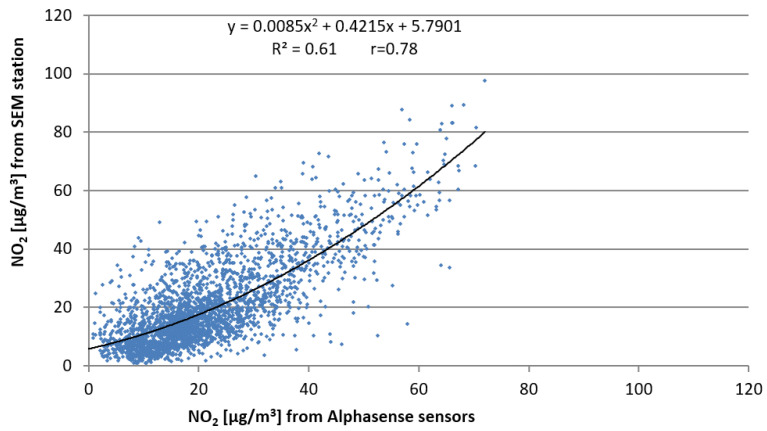
Scatter plot of mean 1 h NO_2_ concentrations from Alphasense sensors compared to the concentrations measured at the SEM station for the initial method (1).

Figure 7 presents that the best fit function was a 2-degree polynomial regression.

The obtained relationship is as follows:

N_A_′ = 0.0085N_A_^2^ + 0.4215N_A_ + 5.7901, (5)

where

- N_A_—NO_2_ concentration measured by the Alphasense sensor, determined from the equation:

N_A_ = (WE_C_ + WE_C_′) s_A_ n_A_, (6)

where

- s_A_—conversion factor (mV) to (ppb) given by Alphasense individually for each sensor;
- n_A_—factor for converting the NO_2_ concentration from (ppb) to (µg/m^3^);
- WE_C_—described by Equation (1);
- WE_C_′—described by Equation (3).

In the last step, multiple regression was used to bind the sensor indications with the meteorological parameters. The independent variables were: 1 h mean temperature (T), 1 h mean relative humidity (H), 1 h mean NO_2_ concentration expressed by the Equation (5), 1 h mean measured voltage (WE_U_ − WE_E_), and the 1 h mean measured voltage (AE_U_ − AE_E_). The dependent variable was the concentration of NO_2_: N_A_″.

Although the Equation (5) is based on the measured WE_U_ and AE_U_ voltages, it was decided to include them as additional, independent variables (or in fact, as differences in relation to the WE_E_ and AE_E_ values) to obtain a relationship with the interval, to which these measured voltages belong (as opposed to Equation (5), where information about the measured voltage values is lost). Secondly, the lack of additional consideration of WE_U_ and AE_U_ voltages led to worse results than those presented below.

The obtained, final relationship for the original method (1) is as follows:

N_A_″ = 1.059N_A_′ − 0.244(WE_U_ − WE_E_) + 0.463(AE_U_ − AE_E_) − 0.304T − 0.023H + 5.54, (7)

**Figure 8:**
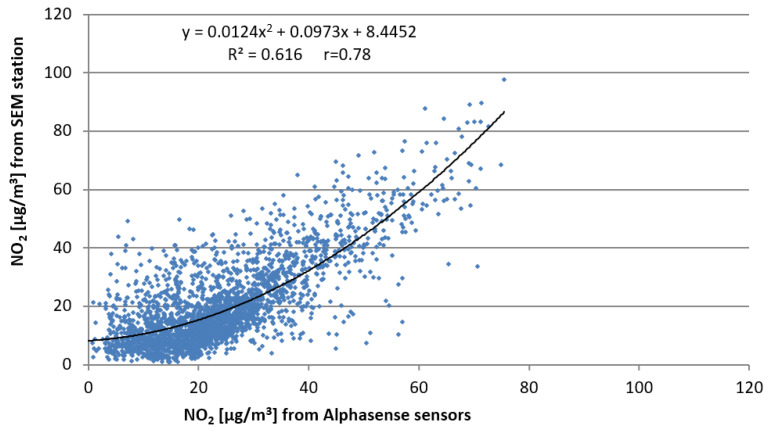
Scatter plot of 1 h mean NO_2_ concentrations from Alphasense sensors compared to the concentrations measured at the SEM station for the initial method (2).

The analysis for the second method (described by the original Equation (2)) was carried out similarly. The best fit (among regression: linear, exponential, polynomial—see Table 5) between the indications of Alphasense sensors and measurements from the SEM stations was obtained by a polynomial regression of the second degree (Figure 8). The obtained relationship is as follows:

N_A_′ = 0.0124N_A_^2^ + 0.0973N_A_ + 8.4452, (8)

After applying multiple regression, the final form of the relationship for the original method (2) is as follows:

N_A_″ = 1.05N_A_′ − 0.087(WE_U_ − WE_E_) + 1.091(AE_U_ − AE_E_) − 0.116T − 0.019H − 3.32, (9)

Statistical parameters after the conversion of the measurement results for NO2_1 and NO2_2 sensors using Equations (7) and (9) are presented in Table 6.

The effectiveness of the determined relationship was checked with the use of a new NO_2_-B43F (indicated later in the text as NO2_3) sensor during the measurement campaign conducted at the Warsaw-Chrościckiego SEM station from December 2019 to May 2020. The results were calculated using Equation (7) and the basic equation recommended by the manufacturer. The results are presented in Table 7.

During the analyzed period, the statistical parameters describing the measurements calculated using method (1) were closer to the results received from the SEM air quality monitoring station than those presented in Table 2, Table 3, Table 4 and Table 5. Therefore, the application of the correction method did not bring such a spectacular improvement as in the measurements in Nowy Sącz. The reason for this could be the fact that in the analyzed period, there were no very high temperatures, as was the case during the measurements carried out between July and September 2019.

Nevertheless, the application of the correction function resulted in the improvement of the measurement data quality. The value of the correlation coefficient improved each month—the highest increase was recorded in May and the lowest in March. In all months, the mean absolute percentage error improved, with the best values obtained for the colder months, and slightly worse for the warmer months. Similarly, to the measurements carried out in Nowy Sącz, and also in Warsaw-Chrościckiego, it can be observed that the sensors during the colder months tend to underestimate the measurements, and during the warmer months—to overestimate (even after applying the correction method).

## 4. Discussion

As the analyzed low-cost NO_2_ sensors at the output do not indicate the measured concentration of the pollutant, Alphasense recommends the use of one of two different methods to determine the voltage. The analysis showed that slightly better results were obtained using method (2)—especially in terms of the mean absolute percentage error. Taking into account the correlation coefficient, in the case of the NO2_1 sensor, the method using the relationship (1) turned out to be better, and in the case of the second sensor—it is difficult to indicate a better variant.

Additionally, none of the methods proposed by the manufacturer offer a satisfactory quality of measurements, as the long-term percentage error maybe even be over 150%, and in certain situations, implementation of the recommended methods may lead to negative concentrations of the pollutant.

The conducted research presented in this paper as well as other studies have shown that high temperature, in particular, is one of the factors interfering with the accuracy indications of Alphasense NO_2_ sensors [24]. As it results from the relationships provided by the manufacturer, to determine the value of the measured pollutant concentration, calculations should be made using the measured voltages and individual values provided for a given sensor by the manufacturer. However, in both Equations (1) and (2) there is also a temperature-related parameter (n_T_ or k′_T_, depending on the method) given by the manufacturer (these values are common to all instances of a given series of sensors). In both relationships, it is used for indications related to the auxiliary electrode, which reduces the temperature influence. However, the values of the n_T_ and k′_T_ coefficients provided by the manufacturer do not completely fulfill their role, because, during high air temperature, the determined NO_2_ concentration turned out to be negative. Therefore, we proposed an additional factor (offset), modifying the corrected WE_C_ voltage. Its role was to react when the temperature exceeds a certain threshold. In the presented analysis, the threshold was determined empirically, observing the temperature above which voltage disturbances appeared on the electrodes, causing a negative concentration.

The result of the application of the proposed coefficient is shown in Figure 6. It shows a significant improvement in the results from Alphasense sensors during periods with high temperatures. First, there were no longer any negative concentrations. Secondly, the corrected values resulted in a significant approximation of the values measured by low-cost sensors to the values determined by the SEM station. This was also reflected in the improved coefficient of determination between the corrected measurement results of the Alphasense sensors and the indications of the SEM stations. The values for the relationships proposed by the manufacturer and after adding the designated offset are presented in Table 8.

**Figure 9:**
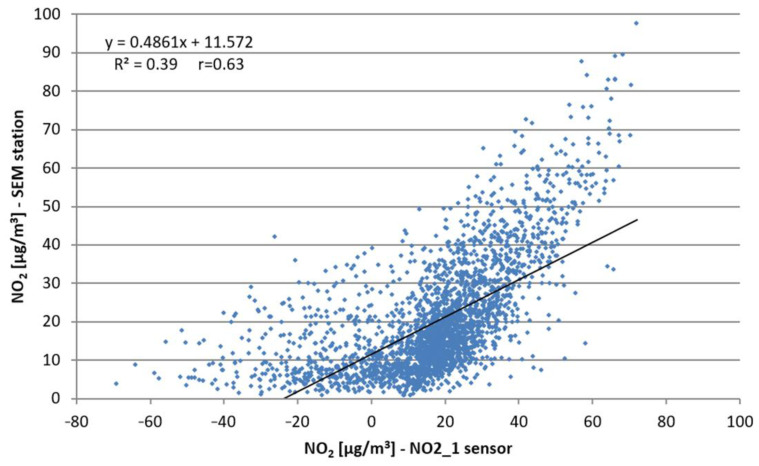
Scatter plot of mean 1 h NO_2_ concentrations from NO2_1 sensor (without correction, with method (1) applied) compared to the concentrations measured at the SEM station.

**Figure 10:**
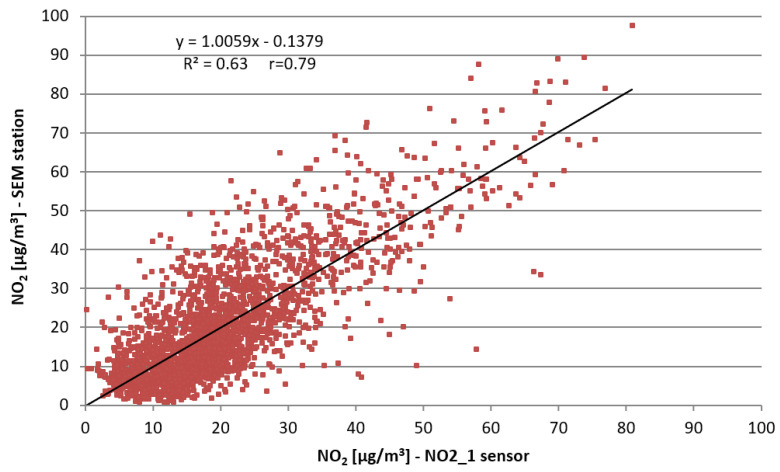
Scatter plot of mean 1 h NO_2_ concentrations from NO2_1 sensor (after correction with Equation (7) applied) compared to the concentrations measured at the SEM station.

The introduced offset did not improve the situation in the remaining period when the temperatures were closer to those recommended by the manufacturer. Taking into account the high correlation of NO_2_ Alphasense sensor indications with the concentrations measured in SEM stations, correction functions were determined for both methods recommended by the manufacturer. Their application in the form of Equations (7) and (9) resulted primarily in the improvement in the correlation coefficients, and the reduction in the mean absolute errors and the mean absolute percentage errors. As is presented in Figure 9 and Figure 10, the implementation of the correction function resulted in the improvement in R^2^ values as follows: R^2^ = 0.39 without correction to R^2^ = 0.63 after correction.

Comparable changes (this means—improvement) in R^2^ values can be found in other research results [20,30], presenting that the correction formula improved the coefficient of determination between analyzed measurement stations from R^2^ = 0.207 to R^2^ = 0.709. The results show also that the sensor’s performance is better in the months with lower temperatures. Although, regardless of the method used—in such months, the correction formula tends to underestimate the measurement values. The opposite trend was visible in other studies. An Alphasense NO_2_ sensor was used in calibration research in Beijing China, where the impact of seasonal changes in its performance was observed [31]. Relative bias was growing with the increase in the temperature. The R^2^ = 0.76 for measurements conducted in the fall decreased to R^2^ = 0.12 in the summer.

## 5. Conclusions

Several months of comparative measurements of Alphasense low-cost NO_2_ sensors with a professional device in various climatic conditions have shown that using the manufacturer’s algorithms, the obtained measurement results are characterized by large measurement errors. It indicated the need to identify factors influencing the measurement values and to mathematically correct the obtained results. In the case of the presented research, the focus was on meteorological factors—air temperature and relative humidity.

Using the collected comparative data, to improve the quality of the measurements, a correction function was proposed that uses raw measurements as well as data on temperature and relative humidity. To verify its effectiveness, several months of comparative measurements were carried out with the use of other sensors and in a different location. This correction function—in many cases—significantly improved the quality of the data. In most cases, after applying the correction, the mean monthly absolute percentage error was reduced to 40–50%. In addition to the reduction in deviations from the actual values, the stabilization of long-term measurement errors was also achieved. Using the original equations recommended by the sensors’ manufacturer, the range of long-term percentage measurement errors was over 100%. The application of the proposed methods reduced the range of these errors to approximately 20%.

The achieved improvement in the quality of measurements may significantly expand the potential applications of this type of sensor. The potential area of application is portable devices that can be easily placed in those locations where, for example, there is a suspicion of high concentrations of pollutants.

However, research on Alphasense sensors cannot be considered complete—the analysis requires, for example, a thorough examination of the scale of the increase in measurement errors with time, or the influence of other pollutants. It would also be desirable to place more sensors in more locations and compare the effectiveness of the proposed algorithm with a professional device. This type of analysis can also be found in other research works.

## Figures and Tables

**Table 1:** n_T_ and k′_T_ coefficient values for NO_2_-B43F sensors provided by the manufacturer.

Temperature (°C)								
	−20	−10	0	10	20	30	40	50
n_T_	1.3	1.3	1.3	1	0.6	0.4	0.2	−1.5
k′_T_	1	1	1	1	1	0.4	−0.1	−4

**Table 2:** Statistical parameters of the NO2_1 and NO2_2 sensors measurement results in particular months of the analyzed period for methods (1) and (2).

	Sensor	Method	July	August	September	October	November
Pearson’s correlation coefficient *r*	NO2_1	1	0.566	0.615	0.727	0.734	0.715
		2	0.561	0.657	0.698	0.702	0.615
	NO2_2	1	0.598	0.679	0.789	0.777	0.657
		2	0.592	0.725	0.745	0.754	0.602
Mean error (µg/m^3^)	NO2_1	1	−3.36	−1.32	−0.65	−4.86	−4.66
		2	−1.04	1.18	2.76	0.46	−0.13
	NO2_2	1	−4.11	−0.02	0.49	−6.01	−7.41
		2	−3.32	0.87	1.85	−2.14	−4.78
Mean percentage error (%)	NO2_1	1	−70.01	−46.21	−8.67	−3.24	1.34
		2	−34.35	−5.49	35.23	33.90	28.09
	NO2_2	1	−62.86	−20.57	7.46	−16.51	−16.88
		2	−41.90	5.09	32.08	9.07	−3.89
Mean absolute error (µg/m^3^)	NO2_1	1	15.13	13.85	8.22	8.78	8.17
		2	12.68	11.96	9.76	9.80	9.30
	NO2_2	1	12.98	12.27	7.64	8.88	10.47
		2	11.04	4.43	8.55	8.32	9.04
Mean absolute percentage error (%)	NO2_1	1	166.16	154.08	93.33	54.64	41.91
		2	134.24	123.00	105.73	72.51	58.48
	NO2_2	1	136.40	119.57	71.21	49.18	52.04
		2	110.83	50.61	83.27	52.84	47.44
Mean square error (µg/m^3^)	NO2_1	1	433.79	342.82	137.24	162.56	123.94
		2	312.02	245.51	144.82	157.02	130.11
	NO2_2	1	308.02	242.484	104.93	160.23	175.44
		2	232.28	61.32	115.70	140.99	166.31

**Table 3:** Mean, maximum, and minimum temperature and air humidity in the chamber of the measuring device and mean NO_2_ concentration from SEM station in particular months of the analyzed period.

	July	August	September	October	November
Mean temperature (°C)	26.5	25.5	19.3	15.0	12.4
Maximum temperature (°C)	41.1	41.7	40.1	33.2	23.3
Minimum temperature (°C)	12.1	14.3	7.5	0.1	0
Mean relative humidity (%)	53.2	55.3	55.9	57.3	61.1
Maximum relative humidity (%)	78.9	81.9	80.2	79	80.1
Minimum relative humidity (%)	18.9	20.3	19.1	21.3	29.2
Monthly mean NO_2_ concentration (µg/m^3^) from SEM station	16.8	16.6	17.9	24.3	23.8

**Table 4:** Statistical parameters of NO2_1 and NO2_2 sensors measurement results in July and August 2019 for the relationships (1) + (3) and (2) + (4).

	Method	NO2_1		NO2_2	
		July	August	July	August
Pearson’s correlation coefficient *r*	(1) + (3)	0.674	0.818	0.671	0.802
	(2) + (4)	0.640	0.797	0.634	0.791
Mean error (µg/m^3^)	(1) + (3)	5.53	6.08	3.83	6.21
	(2) + (4)	4.76	6.21	2.14	2.96
Mean percentage error (%)	(1) + (3)	58.64	73.45	46.23	78.31
	(2) + (4)	51.74	77.15	33.19	50.66
Mean absolute error (µg/m^3^)	(1) + (3)	8.40	8.35	7.48	8.85
	(2) + (4)	8.31	8.84	7.58	4.04
Mean absolute percentage error (%)	(1) + (3)	72.88	85.64	64.86	92.09
	(2) + (4)	71.69	92.08	65.72	56.74
Mean square error (µg/m^3^)	(1) + (3)	116.05	100.79	100.31	114.68
	(2) + (4)	123.62	115.18	105.42	50.69

**Table 5:** Correlation coefficients for different kinds of regressions for Equations (1) and (2).

Kind of Regression	Equation (1)	Equation (2)
Linear	0.76	0.75
Exponential	0.68	0.67
Polynomial	0.78	0.78

**Table 6:** Statistical parameters of the measurement results from the NO2_1 and NO2_2 sensors in particular months of the analyzed period for methods (7) and (9).

	Sensor	Method	July	August	September	October	November
Pearson’s correlation coefficient *r*	NO2_1	7	0.699	0.829	0.858	0.859	0.745
		9	0.681	0.833	0.855	0.878	0.777
	NO2_2	7	0.672	0.803	0.839	0.842	0.697
		9	0.652	0.805	0.829	0.856	0.755
Mean error (µg/m^3^)	NO2_1	7	2.57	3.47	1.25	−3.42	−4.47
		9	1.68	3.03	1.08	−2.80	−3.95
	NO2_2	7	1.19	3.50	2.74	−2.71	−4.74
		9	−0.87	2.16	2.58	−0.98	−2.78
Mean percentage error (%)	NO2_1	7	35.21	50.61	41.41	11.73	7.60
		9	28.65	46.98	40.70	12.62	9.46
	NO2_2	7	23.42	51.06	50.95	11.65	5.47
		9	0.73	31.79	46.43	19.88	12.86
Mean absolute error (µg/m^3^)	NO2_1	7	6.65	6.71	5.34	7.21	8.15
		9	6.41	6.17	5.32	6.63	7.75
	NO2_2	7	6.50	6.71	6.33	6.78	8.52
		9	7.24	6.94	6.44	6.51	7.50
Mean absolute percentage error (%)	NO2_1	7	56.02	68.03	60.03	43.96	42.55
		9	53.84	63.98	59.01	41.43	40.70
	NO2_2	7	52.69	68.37	67.76	42.12	44.99
		9	63.09	63.74	66.85	44.16	41.10
Mean square error (µg/m^3^)	NO2_1	7	78.81	69.44	43.88	92.47	126.12
		9	77.62	67.28	45.76	77.72	114.53
	NO2_2	7	82.78	92.09	62.37	88.65	131.01
		9	97.48	94.12	66.39	76.01	100.37

**Table 7:** Statistical parameters of the measurement results from the NO2_3 sensor in particular months of the analyzed period for Equation (2).

	Method	December	January	February	March	April	May
Pearson’s correlation coefficient *r*	2	0.837	0.541	0.566	0.779	0.785	0.696
	7	0.858	0.617	0.619	0.808	0.865	0.856
Mean error (µg/m^3^)	2	−11.93	−14.09	−11.29	−6.72	0.79	4.17
	7	−6.92	−8.80	−7.05	−4.65	1.40	3.82
Mean percentage error (%)	2	−54.03	−57.79	−41.05	−11.79	26.46	42.44
	7	−8.64	−22.97	−11.38	10.85	24.49	41.17
Mean absolute error (µg/m^3^)	2	11.95	14.43	11.74	9.10	8.25	8.24
	7	8.88	10.07	8.94	8.16	7.86	6.90
Mean absolute percentage error (%)	2	54.20	59.23	46.69	36.40	52.62	61.17
	7	40.01	37.47	35.77	35.73	49.51	51.29
Mean square error (µg/m^3^)	2	218.106	291.33	268.97	177.42	113.25	442.92
	7	74.45	86.47	100.48	69.29	100.39	94.94

**Table 8:** Coefficients of determination (R^2^) for measurements from NO2_1 and NO2_2 sensors in relation to the SEM station without and with offset added for the relationship (1) and (2) for 20 July 2019.

	NO2_1	NO2_2
Method (1) without offset	0.335	0.385
Method (2) without offset	0.304	0.361
Method (1) with offset	0.509	0.664
Method (2) with offset	0.489	0.607

## Data Availability

Measurement data used in the article are publicly available and come from the resources of the Polish Inspectorate for Environmental Protection and are available here: http://powietrze.gios.gov.pl/pjp/archives (access date: 1 February 2022).
